# Oncologic outcome of colon cancer with perforation and obstruction

**DOI:** 10.1186/s12876-022-02319-5

**Published:** 2022-05-15

**Authors:** Kwan Mo Yang, Min-Jae Jeong, Kwang Hyun Yoon, Yun Tae Jung, Jae Young Kwak

**Affiliations:** Department of Surgery, Gangneung Asan Hospital, University of Ulsan College of Medicine, Gangneung, Korea

**Keywords:** Colon cancer, Perforation, Obstruction, Oncologic outcome, Survival

## Abstract

**Purpose:**

Perforation and obstruction in colorectal cancer are poor prognostic factors. We aimed to evaluate the oncological outcomes of patients with colon cancer presenting with perforation or obstruction.

**Methods:**

A total of 260 patients underwent surgery for colon cancer between January 2015 and December 2017. Among them, 54 patients who underwent emergency surgery for perforated (n = 32) or obstructive (n = 22) colon cancer were included.

**Results:**

The perforation (PG, n = 32) and obstruction groups (OG, n = 22) did not differ significantly in age (*p* = 0.486), sex (*p* = 0.821), tumor stage (*p* = 0.221), tumor location (*p* = 0.895), histologic grade (*p* = 0.173), or 3-year overall survival rate (55.6% vs. 50.0%, *p* = 0.784). However, the PG had a higher postoperative complication rate (44% vs. 17%, *p* = 0.025), longer intensive care unit stay (4.8 days vs. 0.8 days, *p* = 0.047), and lower 3-year recurrence-free survival (42.4% vs. 78.8%, *p* = 0.025) than the OG. In the multivariate analysis, perforation was significantly increased risk of recurrence (hazard ratio = 3.67, 95% confidence interval: 1.049–12.839, *p* = 0.042).

**Conclusion:**

Patients with colon cancer initially presenting with perforation had poorer recurrence-free survival, higher postoperative complication rates, and longer ICU stays than those who had obstruction.

## Background

Approximately 15–40% of patients with colorectal cancer present with surgical emergencies, most commonly perforation or obstruction [1–3]. The prevalence of perforation in patients with colorectal cancer is 3–10%, and that of obstruction is 8–20% [1, 2, 4]. Obstruction and perforation caused by colorectal cancer are associated with poor oncologic outcomes and postoperative morbidity [5–8]. In the National Comprehensive Cancer Network guidelines, perforation or obstruction are categorized as high-risk features in colon cancer [9]. Although it is clear that the overall mortality is higher for those treated emergently, it remains unknown whether the surgery predisposes patients to lower long-term survival, even after considering differences in patient characteristics [5]. Indeed, there are only a few studies that directly compare the oncologic outcomes of patients with perforation and those with obstruction. In this study, we aimed to evaluate the oncological outcomes of patients with colon cancer initially presenting with perforation or obstruction.

## Methods

### Patients

Patients who underwent surgery for colorectal cancer at the Gangneung Asan Hospital between January 2015 and December 2017 were enrolled in this study. A total of 367 patients underwent surgery for colorectal cancer during this period. The exclusion criteria were as follows: rectal cancer, iatrogenic perforation during colonoscopy, perforation remote from the primary tumor site, obstruction with successful stent insertion, death within 30 days after surgery, concurrent distant metastasis at diagnosis, concurrent inflammatory bowel disease, hereditary colorectal cancer syndromes, concurrent malignancy, prior history of malignancy, and short follow-up time (< 12 months). The study protocol was approved by the institutional review board of the Gangneung Asan Hospital (registration no: 2021–11-008), in accordance with the Declaration of Helsinki.

**Fig. 1:**
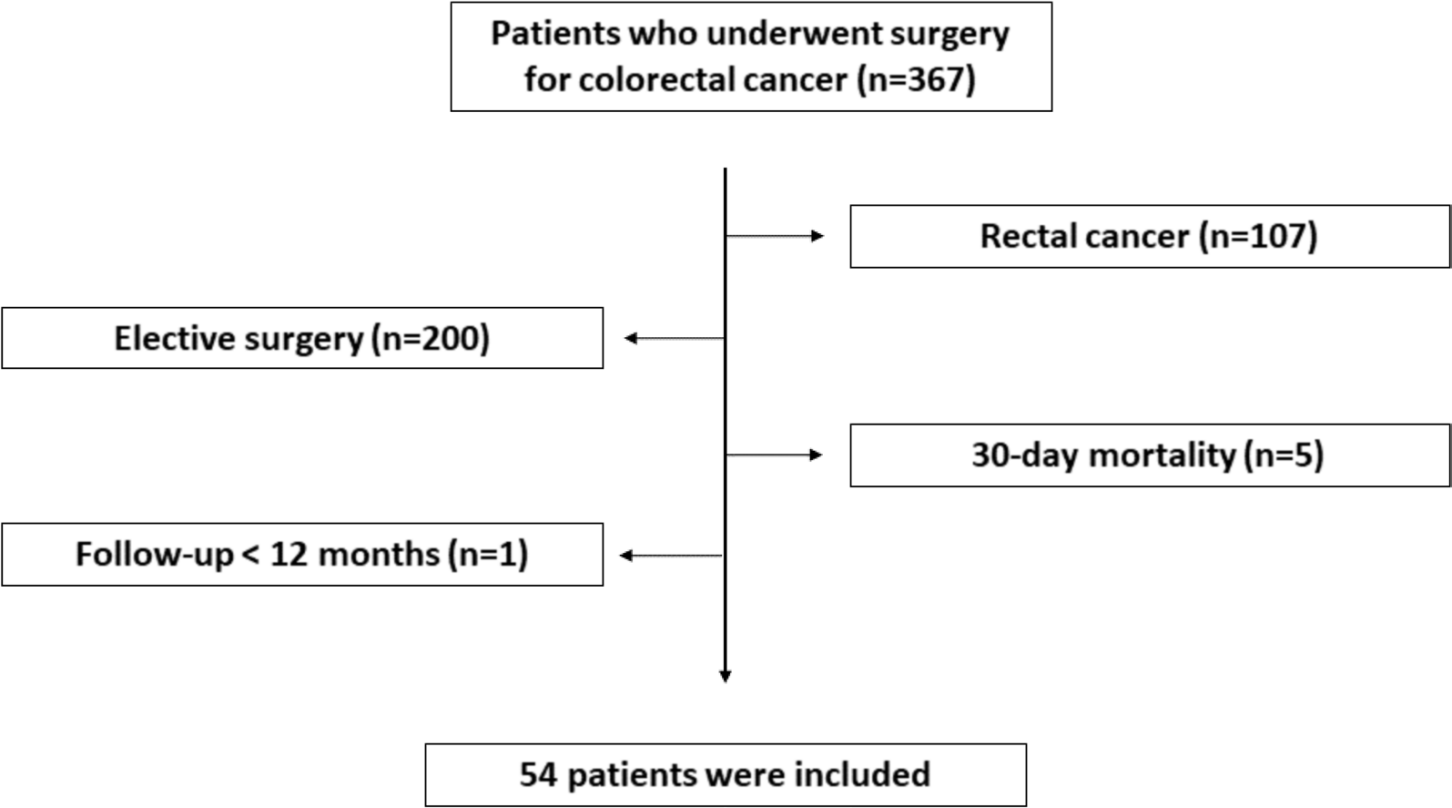
Inclusion criteria

A total of 54 patients were finally included in our analysis (Fig. 1). The patients were divided into two groups according to their initial presenting symptoms. The perforation group (PG, n = 32) included patients with a perforation at the primary cancer site, which was confirmed with operative records. Patients were assigned to the obstruction group (OG, n = 22) through review of the clinical, radiological, and intraoperative findings (complete obstruction). The following patient characteristics were analyzed: age, sex, primary tumor location, pathologic TNM stage, presence of lymphovascular and/or perineural invasion, histologic differentiation, and metastasis type.

For postoperative surveillance, patients were followed routinely at 3- or 6- month intervals for the first 2 years and at 6- or 12- month intervals thereafter. At each visit, Carcinoembryonic antigen (CEA) levels were assessed, a full history was obtained, and a physical examination was performed. Colonoscopy was performed within 6 months to 1 year following surgery and every 2 or 3 years thereafter. Abdominopelvic computed tomography (APCT) and chest computed tomography (CT) were performed 3 or 6 months after surgery and then semiannually for APCT and annually for chest CT. Unscheduled CT or positron emission tomography scans were performed for patients with increased serum CEA concentrations or patients who were symptomatic. KRAS mutation were evaluated using a PNA ClampTM mutation detection kit KRAS (Panagene, Daejeon, Korea).

### Statistical analyses

Survival curves were plotted using the Kaplan–Meier method and compared using the log-rank test. The associations between clinical factors and recurrence-free survival were assessed using the Cox proportional hazard regression model. Comparisons between the PG and OG were performed using the chi-squared test or Fisher’s exact test for categorical variables and Student’s t-test for continuous variables. A p-value < 0.05 was considered statistically significant.

## Results

### Patient characteristics

Except the patients with rectal cancer (n = 107), the perforation and obstruction rates in our study cohort were 12.3% (32/260) and 8.4% (22/260), respectively. Among these 54 patients, 17 (31%) had stage II disease, 23 (43%) had stage III disease, and 14 (26%) had stage IV disease. Twenty-two patients (69%, 22/32) in perforation group and 21 patients (95%, 21/22) demonstrated well/moderate differentiation.

The profile of the patients who were included in this study is shown in Table 1. The PG and OG did not differ significantly in age (*p* = 0.486), sex (*p* = 0.501), tumor stage (*p* = 0.221), tumor location (*p* = 0.895), or administration of adjuvant chemotherapy (*p* = 0.286). However, the PG had a higher postoperative complication rate (44% vs. 17%, *p* = 0.025), a longer intensive care unit (ICU) stay (4.8 days vs. 0.8 days, *p* = 0.047), more poorly differentiated tumors (31% vs. 5%, *p* = 0.019), and a higher tumor recurrence rate (42% vs. 12%, *p* = 0.034). OG showed lower MSH2 positive (77% vs. 97%, *p* = 0.036), MLH1 positive (77% vs. 94%, *p* = 0.071), and higher KRAS mutation (50% vs/ 37%, *p* = 0.222).

**Table 1:** Clinicopathological characteristics

	Perforation (n = 32)	Obstruction (n = 22)	*P*-value
Age, y	67.6 ± 15.2	64.1 ± 15.9	0.486
Sex			0.501
Male	23 (72%)	15 (68%)	
Female	9 (28%)	7 (32%)	
Follow-up, months	26.2 ± 21.6	26.6 ± 19.8	0.892
Location			0.895
Rt sided colon	10 (28%)	6 (25%)	
Lt-sided colon	22 (61%)	16 (67%)	
Stage			0.221
II	13 (41%)	4(18%)	
III	12 (37%)	11 (50%)	
IV	7 (22%)	7 (32%)	
T stage			0.569
3	15 (47%)	14 (64%)	
4a	13 (41%)	6 (27%)	
4b	4 (12%)	2 (9%)	
N stage			0.236
0	14 (44%)	5 (23%)	
1	10 (31%)	11 (50%)	
2	8 (25%)	6 (27%)	
M stage			0.420
0	25 (78%)	15 (68%)	
1a	4 (12%)	5 (23%)	
1b	1 (3%)	0 (0%)	
1c	2 (6%)	2 (9%)	
Retrieved lymph nodes	24 ± 10	24 ± 9	0.560
OP name			0.107
Rt.hemicolectomy	9 (36%)	6 (40%)	
Ant.resection	3 (12%)	1 (7%)	
Low. Ant. Resection	0 (0%	1 (7%)	
Lt. hemicolectomy	1 (4%)	3( 20%)	
Total colectomy	1 (4%)	2 (13%)	
Hartmann's operation	11 (44%)	2 (13%)	
Complication			0.025*
No	18 (56%)	18 (82%)	
Wound complication	7 (22%)	0 (0%)	
Pneumonia	1 (3%)	2 (9%)	
Others	6 (19%)	2 (9%)	
Tumor size, cm	5.5 ± 2.2	5.2 ± 1.6	0.506
Histologic grade			0.019*
Well, moderate	22 (69%)	21 (95%)	
Poor, mucinous, others	10 (31%)	1 (5%)	
LVI, positive	18 (56%)	13 (59%)	0.593
PNI, positive	11 (34%)	10 (45%)	0.746
MLH1, positive	30 (94%)	17 (77%)	0.071
MSH2, positive	31 (97%)	17 (77%)	0.036*
KRAS mutant	12 (37%)	11 (50%)	0.222
ICU stay (Days)	4.9 ± 9	0.9 ± 1.1	0.018*
Adjuvant chemotherapy			0.286
No	14 (44%)	5 (23%)	
Yes	16 (50%)	16 (73%)	
Unknown	2 (6%)	1 (4%)	
Tumor recurrence rate, % (except stage IV)	9/25 (36%)	1/15 (7%)	0.040*
Recurred tumor treatment			0.423
No	1	0	
Chemotherapy	5	0	
Operation	2	1	
Radiation	1	0	
Recurrence sites, % (except stage IV)			0.092
Local	2/25 (8%)	0 (0%)	
Systemic	7/25 (28%)	1 (7%)	
No	16 (64%)	14 (93%)	

Data are presented as n (%) or medians ± standard deviations

*LVI* = lymphovascular invasion, *PNI* = perineural invasion, *ICU* = intensive care unit

Recurrence was occurred in 9 patients in perforation (9/25, 36%) group, and 1 (1/15, 7%) patient in obstruction group. Among them, 5 patients in perforation group were treated with chemotherapy include target therapy, 2 patients received surgical treatment, one patient had radiation therapy, and one patient did not have any treatment due to poor condition.

### Oncologic outcomes according to initial symptoms

**Fig. 2:**
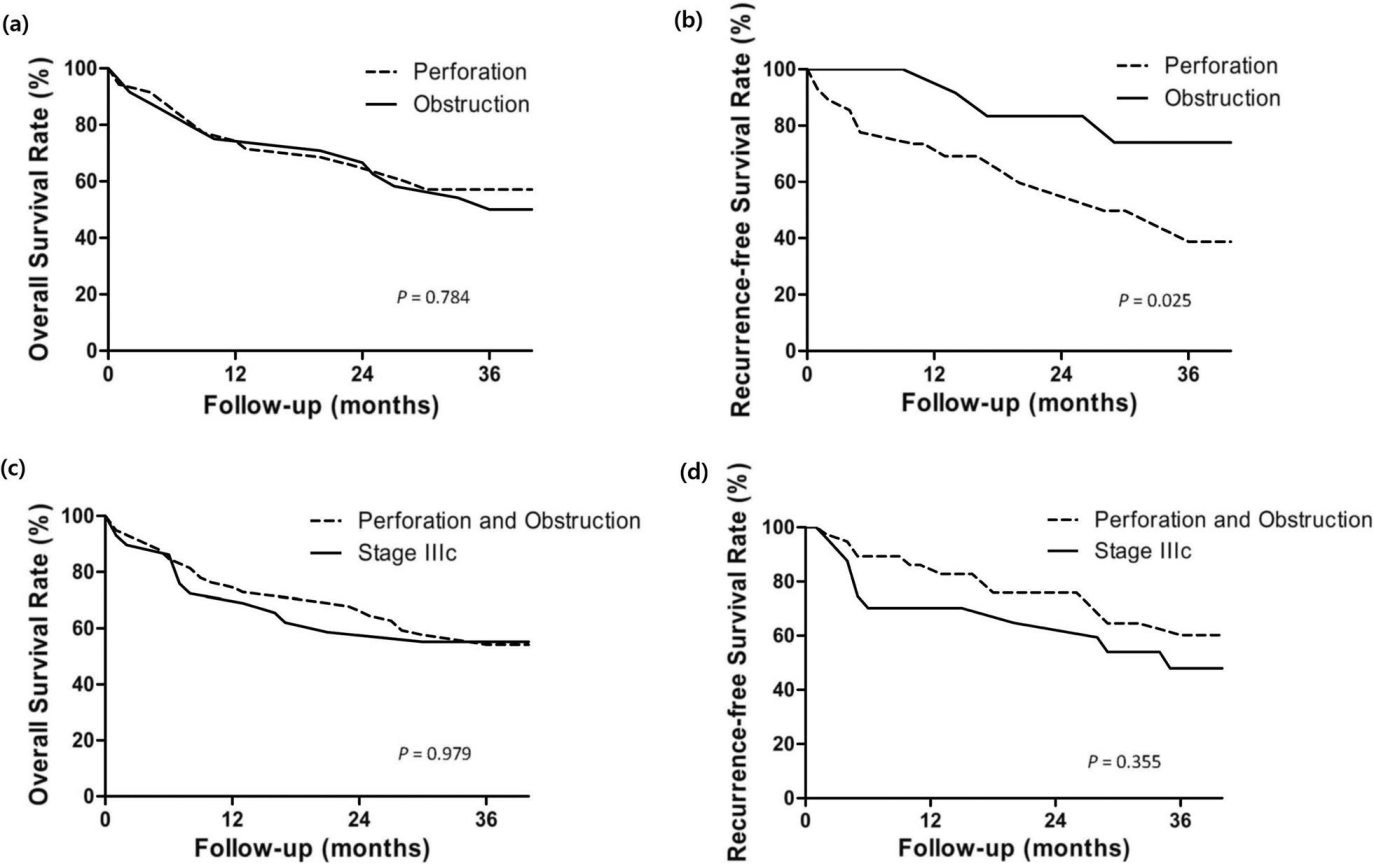
Comparison of overall survival (**a**) and recurrence-free survival (**b**) in the perforation and obstruction groups. Comparison of overall survival (**c**) and recurrence-free survival (**d**) in all patients with colon cancer with perforation/obstruction and patients with stage IIIc colon cancer

The median follow-up duration was 31 months (range: 3–72 months). There was no significant difference in the 3-year OS between the PG and OG (55.6% vs. 50%, p = 0.784). However, the PG did have a significantly lower 3-year recurrence-free survival than the OG (42.4% vs. 78.8%, *p* = 0.025, Fig. 2).

In the multivariate analysis, perforation (hazard ratio [HR] = 4.324, 95% confidence interval [CI]: 1.136–18.688, *p* = 0.041) and presence of perineural invasion (HR = 3.118, 95% CI: 1.441–6.750, *p* = 0.004) were significant risk factors for recurrence-free survival (Table 2). Presence of postoperative complications (HR = 3.809, 95% CI: 1.642–8.840, *p* = 0.002), stage (HR = 3.955, 95% CI: 1.139–13.739, p = 0.018), and presence of perineural invasion (HR = 2.258, 95% CI: 1.090–4.677, *p* = 0.030) were significant risk factors for overall survival (Table 3).

**Table 2:** Univariate and Multivariate analysis of factors associated with Overall Survival (n = 54)

	Univariate		Multivariate	
	*p*-value	HR	95% CI	*p*-value
Age	0.152			
Sex	0.044*			
Type of symptom	0.134			
Perforation				
Obstruction				
Stage	0.002*			0.018*
II				
III				
IV		3.955	1.139–13.739	
Tumor location	0.147			
Rt-sided colon				
Lt-sided colon				
Rectum				
Complication, yes	0.076	3.809	1.642–8.840	0.002*
ICU stay, yes	0.46			
Lymphovascular invasion, yes	0.122			
Perineural invasion, yes	0.092	2.258	1.090–4.677	0.030*
Histologic grade	0.393			
Well to moderate				
Poor to Mucinous				
Adjuvant chemotherapy, yes	0.672			

CI = Confidence interval, HR = Hazard ratio

*p < 0.05

**Table 3:** Univariate and Multivariate analysis of factors associated with Recurrence free Survival (n = 40)

	Univariate		Multivariate	
	*p*-value	HR	95% CI	*p*-value
Age	0.391			
Sex (male)	0.244			
Type of symptom	0.085			0.041*
Perforation		4.324	1.136–18.688	
Obstruction				
Stage	0.708			
II				
III				
Tumor location	0.692			
Rt-sided colon				
Lt-sided colon				
Complication, yes	0.744			
ICU stay, yes	0.106			
Lymphovascular invasion, yes	0.374			
Perineural invasion, yes	0.046*	3.118	1.441–6.750	0.004*
Histologic grade	0.357			
Well to moderate				
Poor to Mucinous				
Adjuvant chemotherapy, yes	0.164			

CI = Confidence interval, HR = Hazard ratio

*p < 0.05

### Survival comparison to patients with stage IIIc colorectal cancer without perforation/obstruction

The 3-year overall survival (53.3% vs. 55.2%, *p* = 0.979) and recurrence-free survival (59.4% vs. 50.5%, *p* = 0.255) of patients with colon cancer with perforation/obstruction (except patients with stage IV disease) and patients with stage IIIc colon cancer were similar (Fig. 2).

## Discussion

Approximately one-third of patients with colorectal cancer have emergent symptoms, and emergency surgery is associated with a high postoperative mortality rate and poor survival [1, 5–8, 10, 11]. Emergency situations are most commonly related to the complications of tumor obstruction or perforation. Many studies have identified a negative impact of colon cancer complications on survival [3–8, 11–15], although most studies included combined heterogeneous emergent situations (obstruction, bleeding, and perforation). Few retrospective studies have directly examined the differences between obstructive and perforated colon cancer [1–3, 12, 16, 17].

The results of the present study confirm a lower disease-free survival rate in multivariate analysis among patients with colon cancer who initially present with perforation than among those who present with obstruction. Many studies report higher recurrence rates in patients who undergo emergency surgery for colorectal cancer (19–45.2% in cases of obstruction and 41.5–56.4% in cases of perforation) [1, 14, 16, 18]. Our study showed that the PG had a higher overall recurrence rate than the OG (Table 1). Although some studies have directly compared the outcomes of patients with colorectal cancer with perforation and obstruction, they included both patients with colon cancer and those with rectal cancer [8, 12, 16, 17]. Further, many studies did not clearly mention whether the perforation occurred at the tumor site or in the proximal bowel. Our study included only patients with colon cancer alone who had bowel perforation at the tumor site. One other study examined patients with colon cancer and reported that the PG had a poorer disease-free survival than the OG and there was no significant difference in the overall survival between the two groups [1]. A few reports compared survival and recurrence in patients with colon cancer, excluding patients with rectal cancer. However, unlike our study, these authors reported no differences in survival or recurrence between the PG and OG [12, 17]. However, both studies included patients who had bowel perforation because of bowel obstruction.

Previous studies have shown that the 30-day mortality rates in patients with colorectal cancer who underwent emergency surgery varied from 8.3 to 20.5% [2, 3, 7, 12–14]. In the present study, the 30-day mortality rate was 8.3%. Five patients died within 30 days, three from sepsis caused by perforation and two from aspiration pneumonia caused by obstruction. Patients who died in the immediate postoperative period were deliberately removed from the survival analysis, as their inclusion constitutes a bias when evaluating long-term oncological results.

Our study revealed that overall survival was not significantly different between the PG and OG. In general, patients with perforation have higher mortality than patients with obstruction because of higher infection rates and severe peritonitis. However, we excluded patients with a follow-up of less than 3 months and those who died within 30 days after surgery, as these patients would have decreased the overall survival rate.

In our study, patients with colon cancer with perforation or obstruction had an overall 1-year survival rate of 77.3% and a 3-year survival rate of 53.3%. We found that the survival curve was very similar to that of all patients with stage IIIc colon cancer (Fig. 2). This is because patients with perforation or obstruction had a higher frequency of postoperative complications, a higher mortality rate after surgery, and a lower rate of receiving adjuvant chemotherapy due to complications than patients with stage IIIc colon cancer.

A potential risk factor for recurrence in patients with colon cancer is lymph node harvesting. Some authors maintain that fewer lymph nodes are retrieved in emergency surgery than in elective surgery [19]. However, in the present study, the mean number of retrieved lymph nodes in both groups was 24. This result may be because 89% of emergency surgeries in our hospital were performed by specialized colorectal surgeons. Similarly, other studies reported the quality of lymphadenectomy in emergency surgery to be similar to that in elective surgery [15, 16, 20].

The limitations of this study included the retrospective nature of the data analysis, the relatively small sample size, and the fact that it was a single-center study. Despite these limitations, our survival estimates are still valuable, since this study had strict inclusion criteria to accurately compare patients with perforated and obstructive colon cancer.

In conclusion, patients with perforated colon cancer had worse recurrence-free survival, higher postoperative complication rates, longer ICU stays, more poorly differentiated tumors, and a higher tumor recurrence rate than patients with obstructive colon cancer. No differences were observed between patients with obstructive and perforated colon cancer in terms of overall survival. Studies with larger series are needed for further investigation.

## Data Availability

The datasets used and/or analysed during the current study are available from the corresponding author on reasonable request.

## Abbreviations

PG: Perforation group
OG: Obstruction group
APCT: Abdominopelvic computed tomography
IUC: Intensive care unit
